# Supplementary material for: Social determinants of health, health behaviors, and general health among colorectal cancer survivors: A cross-sectional study
Source: PLoS One. 2025 Aug 12;20(8):e0329752. doi: 10.1371/journal.pone.0329752 (PMC12342262; doi:10.1371/journal.pone.0329752)
Supplement: Attachment — Submitted filename: Youran Lee. Response to Reviewers.docx [file pone.0329752.s002.docx]

**2Responses to Editor and Reviewer Comments**

**Manuscript ID: PONE-D-24-56636**

**Title: Social Determinants of Health, Health Behaviors, and General Health among Colorectal Cancer Survivors**

**Reviewer 1**

| **Editor Comments** | **Authors’ Response** | **Location of Edited Text** |
| --- | --- | --- |
|  |  |  |
| **Editor Comments:** |  |  |
| 1) In the introduction, positive effects of consumption of fruits and vegetables have been repetitive, describe it once and avoid repetition | Thank you. The repeated sentence has been removed. | Line 64-66 |
| 2) what is the rationale for choosing the 9 chronic conditions? Patients with a chronic condition related to GIT, including crohn's, ulcerative colitis etc. may have poor health outcomes and should be accounted for. | The 9 chronic conditions (i.e., heart attack/myocardial infarction, angina, stroke, asthma, chronic obstructive pulmonary disease [COPD], depression, kidney disease, diabetes, and arthritis) are the specific conditions assessed by the BRFSS survey. |  |
| 3) History of any other type of cancer is a very important covariate that is not being evaluated | Thank you for this comment. We considered adding oyhrt types or number of cancer diagnosis in the initial stages of the analysis. In the BRFSS questionnaire, the section on cancer history asks participants “What type of cancer was it?” However, the BRFSS then focuses on the most recent cancer diagnosis, which in this sample was CRC, thus the information on timing and other cancers was limited in terms of its utility. |  |
| 4) The categorization of comorbidities into 0,1,2+ is not a standard approach. Using a standard comorbidity scale for example, Charlson index will be a good way of categorization | Thank you. We initially considered and evaluated using total number of comorbidities based on the nine comorbidities considered by BRFSS. As expected, the data distribution was severely skewed, with a small number of cases with a value of 3 or greater. Therefore, we used an approach often applied in multiple regression analysis when the predictor (total comorbidities) are non-normally distributed—reducing the number of comorbidities levels to 3-4 ordinal categories. |  |
| 5) A lot of repetition of the same concept/finding through out the paper, avoid repetition to improve clarity for readers | Thank you. We have reviewed the manuscript carefully and removed the redundancies. We also engaged a professional editor to review the manuscript for concision and repetition. | Line 52-87, Line 296-413 |
| 6) It can be logically explained that patients with chronic conditions might have poor health outcomes. It would be good to have a subgroup analysis of SDOH and positive health behaviors on patients with no comorbidities. | Thank you for this suggestion. Many of the positive SDOH were associated with positive health behaviors at a bivariate level. We elected to include total comorbidities as a covariate in all reported analyses so as to focus on the impact of the SDOH on positive behaviors independent of (above and beyond) the relationship with comorbidities. To better understand the relationship of key variables in the conceptual model, we decided that all analyses should control for total comorbidities. |  |
| 7) Future recommendations are focused on the role of nurses. Add more about how doctors can play a part during follow ups | Thank you, we have expanded the discussion to include health care providers (nurses, physician assistants, and physicians). | Line 395-405 |

**Reviewer 2**

| **Editor Comments** | **Authors’ Response** | **Location of Edited Text** |
| --- | --- | --- |
| **Editor Comments:** |  |  |
| 1. The results of the study were obtained using a survey level data. This can lead to several biases due to inherent design of data collection and study design. This is a major limitation of the study and may limit the inferences drawn from the results. | Thank you. We have expanded the Limitation Section to acknowledge that self-report survey information is prone to information bias, recall bias, and selection bias. | Line 354-367, Line 381-382 |
| 2. Several SDOH components were found to be not associated with general health status. This should be addressed in the discussion section along with positive findings. What could be the reasons for such results? | Thank you. We have expanded the Discussion to address SDOH factors not associated with general health status, and potential reasons for the lack of relationship in the clinical population under study. Also, we added justification for those reasons in the Discussion and Limitation Sections | Line 336-353, Line 371-381 |
| **Introduction** |  |  |
| 1. The following statements needs a citation: a. "SDOH, which encompasses factors such as educational and job opportunities, income, housing, transportation, public safety, food security, social inclusion, and nondiscrimination, drives health inequities and impacts general health status." b. These inequities arise from differences in exposure to risk factors such as unhealthy diets and sedentary lifestyles, limited access to preventive measures like chemoprevention and screening, and inadequate follow-up of abnormal test results. | Citations have been added. | Line 60, 74 |
| 2. The authors did a good job of explaining the relationship between SDOH and cancer incidence, and subsequently SDOH, healthy behaviors, and outcomes among colorectal cancer survivors. However, a more succint introduction would improve readability for readers. | Thank you. The Introduction section has been revised to increase concision and reduce redundancy. |  |
| 3. Please reconsider removing sub-headings from the introduction section as per submission guidelines. | Thank you for this suggestion. The sub-headers have been removed. |  |
| **Methods** |  |  |
| 1. The authors identified colorectal cancer survivors through BRFFS database as "patients who self reported a history of colon or rectal cancer". However, is it possible to ascertain whether these patients are survivors or under treatment or have a recurrence of the disease? This is an important aspect as it has a significant role in how SDOH impacts health outcomes. | The clinical population for the analysis was colorectal cancer (CRC) survivors. The sample included those with a diagnosis of CRC who completed the BFRSS survey (survivors). The BRFSS does include an item asking whether the individual is currently receiving treatment for cancer but does not specifically ask about CRC treatment, thus we did not include the variable in the analysis. There also is no item asking about recurrence. |  |
| 2. Since the data related to positive health behaviors was self reported by patients using telephonic encounter. This data is variable and subject to bias depending on patient's recent beahviors. This is a subjective assessment and may significantly impact the results of current study. | Yes, the BRFSS data were collected through telephone interviews, which is one of the most prevalent methods for gathering population-level health data across the United States. Although self-reported data (particularly in relation to positive health behaviors) may be subject to recall bias and influenced by recent behaviors, this is a common and recognized limitation of survey-based datasets. Nonetheless, the BRFSS remains a valuable and widely used resource for public health surveillance and research. |  |
| 3. The authors reported poor/fair health outcome (0=no, 1=yes) as a categorical variable derived from a 5-point Likert-scale. Such data recoding might cause issues with data analysis as data collection / organized may not be intended for such recategorization / analysis. | Yes, the recoding of the self-rated general health status outcome into a binary outcome potential yields a loss of information. However, the dichotomization of the original 5-point Likert outcome is commonly applied in health science due to severe skewness of the data distribution. Published studies have consistently shown that the dichotomized outcome is useful in differentiating the impact of social factors on general health; therefore, we decided to apply the binary version of this health outcome as operationally defined in a large number of other published secondary analyses using BRFSS data. |  |
