# Supplementary material for: Social determinants of health, health behaviors, and general health among colorectal cancer survivors: A cross-sectional study
Source: PLoS One. 2025 Aug 12;20(8):e0329752. doi: 10.1371/journal.pone.0329752 (PMC12342262; doi:10.1371/journal.pone.0329752)
Supplement: Attachment — Submitted filename: Response to Reviewers.docx [file pone.0329752.s003.docx]

**Responses to Editor and Reviewer Comments**

**Manuscript ID: PONE-D-24-56636**

**Title: Social Determinants of Health, Health Behaviors, and General Health among Colorectal Cancer Survivors**

**Reviewer 3**

| **Editor Comments** | **Authors’ Response** | **Location of Edited Text** |
| --- | --- | --- |
|  |  |  |
| **Editor Comments:** |  |  |
| 1) The use of 2021 BRFSS data, which coincides with the COVID-19 pandemic, may have significantly influenced self-reported health status, health behaviors, and access to care. The potential impact of the pandemic on the findings should be acknowledged and briefly discussed in the limitations section. | Thank you for your feedback. I address this context in the discussion section. | Line 363-367 |
| 2) A prior reviewer noted that the set of chronic conditions included in the analysis omits gastrointestinal (GIT)-related conditions, which may be particularly relevant in a CRC survivor population. While the authors clarified that they relied on the nine chronic conditions available in the BRFSS dataset, this limitation should be explicitly acknowledged in the Methods or Discussion section. | Thank you for your feedback. I address this context in the discussion section. | Line 367-370 |
